# Seroprevalence of *Toxoplasma gondii* infection and risk factors in domestic sheep in Henan province, central China

**DOI:** 10.1051/parasite/2016064

**Published:** 2016-11-24

**Authors:** Nian Zhang, Shuai Wang, Dong Wang, Chaoying Li, Zhenchao Zhang, Zhijun Yao, Tingting Li, Qing Xie, Shiguo Liu, Haizhu Zhang

**Affiliations:** 1 School of Basic Medical Sciences, Xinxiang Medical University Xinxiang Henan 453003 PR China; 2 Xinxiang Medical University Xinxiang Henan 453003 PR China; 3 The Fourth Clinical College, Xinxiang Medical University Xinxiang Henan 453003 PR China; 4 Xinxiang Assegal Medical Examination Institute Xinxiang Henan 453003 PR China

**Keywords:** *Toxoplasma gondii*, seroprevalence, sheep, MAT, central China

## Abstract

Sheep are highly susceptible to infections with *Toxoplasma gondii* and play a major role in the transmission of toxoplasmosis to humans. In the present study, 779 serum samples from sheep were collected from Henan province, central China from March 2015 to May 2016, and antibodies to *T. gondii* were detected by modified agglutination test (MAT). The overall seroprevalence of *T. gondii* in sheep was 12.71% (99/779). The risk factors significantly associated with *T. gondii* seroprevalence were the geographical origin, age, presence of cats, and the rearing system. This is the first report of *T. gondii* infection in sheep in Henan province, central China, and of an association of seropositivity to *T. gondii* with risk factors.

## Abstract (fr)

Les moutons sont très sensibles aux infections par *Toxoplasma gondii* et jouent un rôle majeur dans la transmission de la toxoplasmose aux humains. Dans la présente étude, 779 échantillons de sérum de moutons ont été prélevés dans la province du Henan, en Chine centrale, de mars 2015 à mai 2016, et les anticorps contre *T. gondii* ont été détectés par le test d’agglutination modifié (MAT). La séroprévalence globale de *T. gondii* chez le mouton était 12.71 % (99/779). Les facteurs de risque significativement associés à la séroprévalence anti-*T. gondii* étaient l’origine géographique, l’âge, la présence de chats et le système d’élevage. Ceci est le premier rapport concernant l’infection par *T. gondii* chez le mouton dans la province du Henan, en Chine centrale, et l’association entre la séropositivité à *T. gondii* et les facteurs de risque.

## Introduction

*Toxoplasma gondii* is an important protozoan parasite found worldwide that potentially infects all warm-blooded vertebrates, including mammals, birds, and humans [9, 19, 39]. Sheep are an important intermediate host of *T. gondii* [36]. Infection of sheep with *T. gondii* may cause early embryonic death and resorption, fetal death and mummification, abortion, stillbirth, and neonatal death [6, 14, 32], and thus can be responsible for severe economic losses in the sheep industry.

It has been estimated that up to one third of the world’s human population has been infected by *T. gondii* [38, 40]. Humans become infected postnatally, mainly by ingesting tissue cysts from undercooked meat or from food or drink contaminated with oocysts shed in cat feces [22, 34]. Ingestion of poorly cooked meat from sheep, and possibly consumption of non-pasteurized contaminated milk from sheep are considered important sources of transmission of *T. gondii* to humans [4, 7, 18, 21, 27]. People in Henan province have the habit of eating undercooked “barbecue”, “kabob”, and “instantly boiled mutton”, leading to an increased risk of human toxoplasmosis.

There have been a number of surveys of *T. gondii* infection in sheep in many countries [8, 16, 26, 33]. In China, several surveys have shown that infection of sheep with *T. gondii* is common in other provinces (Table 1; [25, 42, 45]), but there have been no reports of *T. gondii* infection in sheep in Henan province in central China.

**Table 1.** The prevalence of *Toxoplasma gondii* infection in sheep in the People’s Republic of China.

Provinces/Cities	Year of sampling^A^	Age of sheep	No. tested	No. positive	Prevalence (%)	Method^B^	Reference
Heilongjiang	2008–2010	Not shown	792	24	3.0	IHA	[37]
Liaoning	2011	At all ages	566	25	4.4	IHA	[43]
Jinzhou	2012	At all ages	402	72	17.9	MAT	[42]
Qinghai	2012–2013	≥1 year	600	128	21.3	ELISA	[25]
Yunnan	2012–2013	At all ages	154	15	9.7	IHA	[45]
Gansu	2013–2014	At all ages	1732	352	20.3	MAT	[44]

A Years of sampling are listed as published in the references.

B IHA: indirect hemagglutination test; MAT: modified agglutination test; ELISA: enzyme-linked immunosorbent assay.

Therefore, the objectives of the present study were to evaluate the seroprevalence of *T. gondii* infection in sheep, and to identify certain risk factors associated with the prevalence of *T. gondii* infection in Henan province, central China.

## Materials and methods

### Ethics statement

The study was reviewed and approved by the Ethics Review Committee of the Xinxiang Medical University (Reference No. 2015016).

### The study site

The study was conducted in Henan province, located in the central part of mainland China, covering an area of 167,000 km^2^ and having a population of approximately 106 million. Its geographical position is at east longitude 110°21′–116°39′ and at north latitude 31°23′–36°22′. The Yellow River passes through central Henan. The area has a continental monsoon climate, with four distinctive seasons. The average annual temperature is 12.1–15.7 °C, with a mean annual rainfall of 532.5–1380.6 mm. There are 17 provincial cities distributed in Henan province, with the city of Zhengzhou as its capital. Three cities, including Zhoukou (33°03′–34°20′ N, 114°05′–115°39′ E), Zhumadian (32°18′–33°35′ N, 113°10′–115°12′ E), and Xinxiang (35°18′ N, 113°54′ E), were selected for sample collections. All of the above places are the main supply areas for ovine meat to Henan and the neighboring regions.

### Sample collection

A total of 779 blood samples from sheep were collected in the above three cities in Henan province from March 2015 to May 2016. Data regarding age, sex, location, presence of cats, and the rearing system of each animal were recorded. Blood samples were centrifuged and sera were recovered and transferred to 1.5 mL Eppendorf tubes. Subsequently, all the sera were stored at –80 °C until testing for anti-*T*. *gondii* antibodies.

### Determination of antibodies against *T*. *gondii*

Anti-*T. gondii* antibodies were detected in serum samples by the modified agglutination test (MAT), as described previously [1, 11, 44]. *T. gondii* whole cell antigen (formalin-treated tachyzoites) was purchased from KeraFAST, Inc. (Boston, MA, USA) and was used to detect *T. gondii* antibodies in deer [30] and chickens [15] by MAT. This antigen was prepared using the RH strain of *Toxoplasma* cultivated via human foreskin fibroblast cells in culture and the collected tachyzoites were killed by treatment with 6% formaldehyde for at least 16 hours. In brief, twofold dilutions of sera from 1:25 to 1:3200 were performed using the serum diluting buffer, and agglutination was performed in U-bottom 96-well microtiter plates using a mixture of 50 μL antigen and 50 μL diluted sera. The plates were incubated at 37 °C overnight. The test was considered positive when a layer of agglutinated parasites was formed in wells at dilutions of 1:25 or higher based on previous studies [2, 8]. Positive and negative controls were included in each test.

### Statistical analysis

Differences in *T*. *gondii* prevalence with different variables such as age, sex, and presence of cats were analyzed using a chi-square test. Statistical analysis was performed using SPSS 20 software for Windows (SPSS Inc., Chicago, Illinois, USA). The differences were considered statistically significant if *p* < 0.05.

## Results and discussion

The modified agglutination test (MAT) is a sensitive and specific method for the detection of *T. gondii* antibodies in a wide range of host species, and is simple, rapid, and of relatively low cost compared to other serological tests [5, 12, 13, 28]. It has been evaluated extensively in experimentally and naturally infected sheep [2, 8, 28]. Hence, in the present study, we used the MAT to determine the seroprevalence of *T. gondii* in sheep.

As shown in Table 2, the overall seroprevalence of *T. gondii* in sheep in Henan province, central China was 12.71% (99/779). Compared with other provinces in China, the prevalence of 12.71% in sheep was lower than the prevalence of 20.3% in Gansu [44], and 21.33% in Qinghai [25], but higher than the reported prevalence of 3.0% in Heilongjiang [37], 4.4% in Liaoning [43], and 9.7% in Yunnan [45]. These differences may be due to different ecological conditions, climates, serological techniques used, survey periods, sample sizes, and the breed of sheep.

**Table 2.** *Toxoplasma gondii* infection in 779 sheep in Henan province, central China.

Variable	No. tested	No. positive	Prevalence (%)#	95% CI
Region				
Zhoukou	255	25	9.80^A^	6.15–13.45
Zhumadian	272	20	7.35^A^	4.25–10.45
Xinxiang	252	54	21.43^B^	16.36–26.50
Sex				
Male	292	34	11.64	7.96–15.32
Female	487	65	13.35	10.33–16.37
Age (years)				
≤1	246	16	6.50^A^	3.42–9.59
1 ~ 2	292	37	12.67^B^	8.86–16.49
≥2	241	46	19.09^C^	14.13–24.05
Presence of cats				
No	420	31	7.38^A^	4.88–9.88
Yes	359	68	18.94^B^	14.89–23.00
Rearing system				
IR	221	14	6.33^A^	3.12–9.55
SIR	343	43	12.54^B^	9.03–16.04
Extensive	215	42	19.53^C^	14.24–24.83
Total	779	99	12.71	10.37–15.05

# Values with different superscript (A, B, C) in the same column are significantly different (*p* < 0.05).

Geographically, there are 17 provincial cities distributed in Henan province and three places were selected for screening *T. gondii* seroprevalence because they were the main supply areas of mutton. The present study showed that geographical origin represents another risk factor. Seropositive animals from different cities were: 9.80% of 255 from Zhoukou, 7.35% of 272 from Zhumadian, and 21.43% of 252 from Xinxiang. The samples collected from Zhoukou and Zhumadian were less likely to show seropositivity compared to those collected from Xinxiang (*p* < 0.05; Table 2). This difference may be related to the rearing system in these regions. Most sheep were raised semi-intensively or intensively in Zhoukou and Zhumadian, while extensive and semi-intensive rearing systems were widely used for these small ruminants in Xinxiang.

In the present study, the seroprevalence of *T. gondii* in males was 11.64% (34/292) and in females 13.35% (65/487; Table 2). Although the seroprevalence in females was higher than in males, the difference was not significant (*p* > 0.05). This was consistent with previous reports [3, 20, 41, 45].

Furthermore, the seroprevalence of *T. gondii* infection obtained in the present study in sheep increased significantly (*p* < 0.05) with increasing age. The highest prevalence of infection (19.09%) was detected in two-year-old or older animals, followed by intermediate prevalence (12.67%) in the 1–2 year age group, while the prevalence found in animals in the ≤1 year age group was 6.50% (Table 2). These results are similar to those of previous investigations [17, 20, 41, 42], suggesting the possibility of horizontal transmission in the investigated herds.

In the present study, the seropositive rate for *T. gondii* of sheep raised on the farm with the presence of cats was higher than that for animals raised on the farm without cats. This finding is also consistent with previous reports [3, 10, 25]. Liu et al. reported that the odds of the presence of *T. gondii* antibodies increased 3.2-fold if cats were present on the farms [25]. Felids are the only known definitive host of *T. gondii*, and primary infected cats shed millions of oocysts into the environment [24, 29]. Oocysts excreted by cats remain infective for months to years in moist, shaded, and temperature-regulated environmental conditions [23, 35]. The association with the presence of cats is therefore assumed to indicate a causal relationship, and limiting the number of cats on sheep farms is expected to reduce *T. gondii* infections in these small ruminants.

In addition, the seroprevalence obtained in the present study in intensively raised sheep was statistically lower (*p* < 0.05) than that in extensively and semi-intensively raised animals (Table 2). Our findings are similar to those of previous reports [31, 42]. The main reason for such a difference may be that, compared with extensively or semi-intensively raised animals, intensively raised sheep are caged and thus have less chance of ingesting the oocysts of *T. gondii* excreted by infected cats.

## Conclusions

The results of the present survey indicate that *T. gondii* infection is highly prevalent in sheep in Henan province, China. The risk factors significantly associated with *T. gondii* seroprevalence were age, the presence of cats, and the pasturing system on the farms. Integrated and efficient measures are required to prevent and control *T. gondii* infection in sheep in Henan province, China.

## Conflict of interest

We declare that we have no conflict of interest.
